# Eyes-closed finger-tapping task for assessing visual dependence in temporal motor control in Parkinson’s disease

**DOI:** 10.3389/fneur.2026.1887146

**Published:** 2026-09-29

**Authors:** Kazuhide Seo, Masayuki Hara, Masahito Miki, Toshimasa Yamamoto, Genko Oyama

**Affiliations:** 1 Department of Neurology, Saitama Medical University, Saitama, Japan; 2 Graduate School of Science and Engineering, Saitama University, Saitama, Japan

**Keywords:** bradykinesia, finger tapping, motor automaticity, motor symptoms, Parkinson’s disease, visual dependence

## Abstract

**Background:**

Impaired motor automaticity in Parkinson’s disease (PD) may increase reliance on visual feedback, but its role in stabilizing repetitive movements remains unclear.

**Objective:**

To quantify the effect of eye closure on finger tapping and identify performance features reflecting visual dependence in PD.

**Methods:**

Forty-five individuals with PD (74.0 ± 6.6 years; 21 women) and 20 age-matched healthy controls (HC; 74.6 ± 5.5 years; 13 women) performed 10-s index finger–to–thumb tapping under eyes-open and eyes-closed conditions. A wearable device recorded metacarpophalangeal flexion–extension angles at 1 kHz, with 30-Hz low-pass filtering. Six metrics were computed: the mean and variability of amplitude, velocity, and inter-peak interval. Eye-closure effects were estimated as r = ln(closed/open) and analyzed using a linear mixed-effects model with group, side, age, and sex as fixed effects and subject as a random intercept.

**Results:**

A nominal group effect was observed only for interval variability (*p* = 0.0337), whereas no group effects were observed for the other metrics. Eye closure was associated with a numerical increase in interval variability in the PD group (*r* > 0) and a numerical decrease in the HC group (r < 0); however, the corresponding within-group comparisons did not reach statistical significance (PD: *p* = 0.077; HC: *p* = 0.081).

**Conclusion:**

The opposing numerical changes in tapping-interval variability in the PD and HC groups raise the possibility that visual input may contribute differently to temporal stability during repetitive movements in the two groups, warranting further investigation.

## 1 Introduction

Bradykinesia is a cardinal motor symptom of Parkinson’s disease (PD), characterized by slowness of movement, often accompanied by hypokinesia, defined as reduced movement amplitude (1). Impaired motor automaticity is an important pathophysiological basis for bradykinesia (2). Motor automaticity refers to the ability to perform movements smoothly without conscious attention to the details of the movement, particularly during repetitive simple actions and habitual daily activities (3). Even complex movements can become automatic through sufficient training (4). Indeed, many everyday behaviors rely on automatic movements. For example, people usually continue walking smoothly without explicitly attending to the act of walking.

Finger tapping is a standard clinical task for assessing bradykinesia in PD [i.e., Item 3.4 in the Movement Disorder Society-sponsored revision of the Unified Parkinson’s Disease Rating Scale (MDS-UPDRS)] (5) and has been validated extensively in objective quantitative assessments (6). The finger tapping task in the MDS-UPDRS is a simple repetitive movement that can be used to assess motor automaticity while minimizing the effects of postural control and whole-body coordination. In PD, impaired motor automaticity leads to poorer performance in simple repetitive movements, such as finger tapping. Complex movements that were previously learned and automatized can also be impaired, presenting clinically as micrographia (7). Furthermore, because movement execution requires increased attentional resources, symptoms worsen when cognitive resources are taxed by dual-task demands (8–10). External visual cues can improve the motor performance in some cases (11). Functional neuroimaging studies have shown that the basal ganglia–premotor–cerebellar network, which is typically engaged during automated movements, does not operate efficiently in PD and that a compensatory shift toward attentional control mediated by frontal and attentional networks occurs (2, 12, 13). Consequently, simple repetitive movements and habitual daily actions that are normally performed automatically may become more dependent on external visual cues in PD (2). Thus, visual information may serve as a compensatory mechanism to maintain motor automaticity in patients with PD. However, studies directly examining the role of visual information in compensating for reduced motor automaticity are limited.

We hypothesized that visual information plays an important role in maintaining motor automaticity in patients with PD. To evaluate this hypothesis, a wearable exoskeleton-based motion tracking device for index finger movements was developed for the reliable and quantitative assessment of MDS-UPDRS-related finger-tapping metrics, such as amplitude and velocity. Using the device and these metrics, we determined the influence of visual input on finger-tapping performance in patients with PD and age-matched healthy controls (HCs) under eyes-open and eyes-closed conditions. We expected that the decrease in finger-tapping performance would be greater in the eyes-closed condition than in the eyes-open condition in patients with PD. This study contributes to a better understanding of the role of visual information in compensating for impaired motor automaticity in PD and provides useful insights for the development of visual cue-based assessment and rehabilitation strategies.

## 2 Materials and methods

### 2.1 Participants

Forty-five individuals with PD (mean age = 74.0 ± 6.6 years; 21 women) and 20 HCs (mean age = 74.6 ± 5.5 years; 13 women) participated in this study. All participants provided written informed consent before participating in the study. The experimental protocol was approved by the Institutional Review Board of Saitama Medical University (No. 2021–085) and was conducted in accordance with the Declaration of Helsinki.

Patients with PD were recruited from the outpatient clinic at Saitama Medical University Hospital. Inclusion criteria were clinically established PD according to the Movement Disorder Society clinical diagnostic criteria (14), Hoehn and Yahr stage I–III, age 50–90 years, and reduced dopamine transporter uptake on dopamine transporter single-photon emission computed tomography. Exclusion criteria were a history of heavy alcohol use, prior chemotherapy, psychiatric illness, neurodegenerative disorders other than PD, intracranial lesions such as cerebral infarction, intracerebral hemorrhage, or head trauma with loss of consciousness, peripheral neuropathy, cervical spinal cord disorders, severe or unstable medical illness requiring hospitalization, sensory disturbance, muscle weakness, or any other condition judged by the investigators to make the participant unsuitable for the study.

Healthy controls were recruited from patients’ family members and individuals attending the outpatient clinic for conditions other than neurological or psychiatric disorders. Eligible controls were 50–80 years of age and had no evidence of neurological or psychiatric disease based on clinical interview and medical history. The exclusion criteria for the control group were the same as those for the PD group, except for the diagnosis of PD.

### 2.2 Apparatus and data recording

A wearable exoskeleton-based finger tapping measurement device was developed to measure the flexion–extension angle of the metacarpophalangeal (MCP) joint of the index finger during repetitive index-finger-to-thumb tapping tasks (see Figure 1). The device was designed to quantify the primary repetitive flexion–extension component of finger tapping; therefore, rotational and abduction/adduction components were not measured in the present study. Specifically, the rotational part attached to the radial side of the proximal and middle phalanges of the index finger was designed to rotate freely around the axis of a miniature rotary potentiometer (RDC506002A; ALPS Electric Company, Ltd., Tokyo, Japan) fixed to the main body of the device in response to flexion–extension movements. Because the rotational part could move up and down slightly relative to the main body, the rotational axis was flexibly adjusted for each participant such that it was approximately aligned with the MCP joint flexion–extension axis. The main body of the device was made of acrylonitrile butadiene styrene (ABS) and was designed to be easily attached to and removed from the participants’ hands using a hook-and-loop fastener. The device weighed approximately 35 g and therefore was expected to have a negligible influence on task performance.

**Figure 1:**
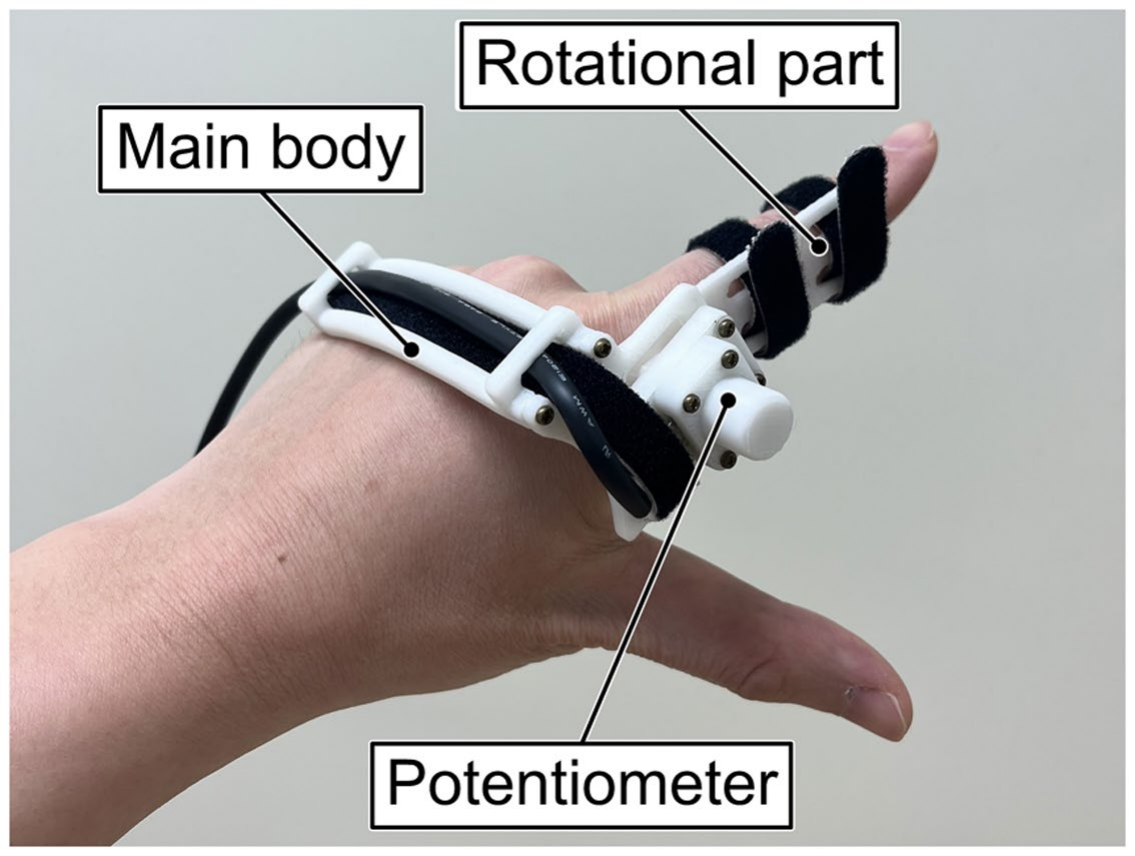
Finger-tapping measurement device. A miniature rotary potentiometer, integrated into the main body of the device and linked to a rotational part, measures the MCP joint flexion–extension angle (defining the thumb-to-index finger contact posture at the start of tapping as 0°) during repetitive index finger-to-thumb tapping tasks. The device was 3D-printed to minimize weight and was designed for easy attachment to and removal from the participants’ hands using a hook-and-loop fastener.

For data recording, the MCP joint flexion–extension angle, defined as the sagittal plane angle between the axes of the second metacarpal and the proximal phalanx, was acquired as an analog voltage signal using a data acquisition device (NI USB-6001; National Instruments, Austin, TX, USA) at a sampling rate of 1 kHz. Using a laptop computer, the analog voltage was converted back into joint angle data, and a low-pass filter (LPF) with a cutoff frequency of 30 Hz was applied to remove noise unrelated to the index-finger tapping movements, typically below 3–5 Hz. For the LPF, a first-order IIR filter was used, and prewarping was employed to compensate for the frequency warping caused by the bilinear transform. The index finger-to-thumb contact posture at the start of tapping was defined as zero, and extension was assigned a positive value. Custom software for the device was developed to allow the experimenter to set the experimental parameters, monitor finger movements both numerically and graphically, and save experimental data through a dedicated graphical user interface. The noise-filtering procedures and software, together with data acquisition, were implemented in Microsoft Visual Studio 2008.

### 2.3 Experimental methods and conditions

All participants performed index finger-to-thumb tapping with both hands under two visual conditions: eyes open and eyes closed (two sides × two visual conditions per participant). As in the clinical assessment of finger tapping in the MDS-UPDRS (5), participants were instructed to tap as widely and as quickly as possible. The task was performed in a fixed order: right hand followed by left hand, and eyes-open condition followed by eyes-closed condition. For each condition, one practice trial was conducted before the test trial, and one test trial was performed for each condition. In patients with PD, all assessments were performed in the ON-medication state.

### 2.4 Outcome measures

In routine clinical settings, movement disorder specialists assess finger tapping performance using the MDS-UPDRS Part III, Item 3.4 (Finger Tapping), with a particular focus on amplitude, velocity, and rhythm (5). In line with the MDS-UPDRS-based assessment, six outcome measures (i.e., mean amplitude, amplitude variability, mean velocity, velocity variability, mean interval, and interval variability) were assessed in the present study. Tapping Count was additionally recorded and reported descriptively in Supplementary File S2 but was not included in the primary analysis, which focused on these six MDS-UPDRS-based outcome measures.

### 2.5 Preprocessing and feature extraction

For each 10 s tapping trial, the time–angle signals were processed using Python. The original sampling frequency was estimated from the mean of the positive time differences in the Time[s] column.

Peak detection on the angle signal θ(t) was performed using scipy.signal.find_peaks, setting the minimum inter-peak distance to 0.1 s. The minimum distance was converted into samples based on the estimated sampling frequency. The effective height (prominence) of each candidate peak was calculated using the SciPy function signal.peak_prominences, and only peaks with a prominence ≥ 0.03 rad were retained for further analyses. The amplitude of each tap was defined as the prominence of the corresponding peak, and its mean and standard deviation (SD), computed using numpy.mean and numpy.std, were considered as the Mean Amplitude (rad) and Amplitude Variability (rad), respectively.

Angular velocity, v(t) = dθ/dt, was obtained by numerical differentiation of the angle signal with respect to the recorded time axis using numpy.gradient(angle_signal, time). The mean of ∣v(t)∣ was defined as Mean Velocity (rad/s), and the SD of the signed velocity v(t) as Velocity Variability (rad/s). Inter-peak intervals were computed from successive peak timestamps using numpy.diff, and the mean and SD of these intervals were defined as the Mean Interval (s) and Interval Variability (s), respectively. The number of detected peaks within each 10-s test trial was recorded as Tapping Count and included in Supplementary File S2 (tapping_trial_metrics.csv) to document the number of taps and corresponding inter-peak intervals available for the interval-based estimates. The abduction/adduction and rotational components of the finger movements were not analyzed. Data import and export were handled using the pandas package (pandas.read_csv, DataFrame.to_csv). The full preprocessing and feature extraction pipeline described above was implemented in a standalone Python script (tapping_analysis_core.py), which is provided as Supplementary File S1, together with documentation (README.md).

#### 2.5.1 Computational environment

All computations were performed in double-precision floating point using Python 3.13.9,^1^ NumPy 2.1.3,^2^ SciPy 1.15.3,^3^ and pandas 2.2.3.^4^

### 2.6 Dependent measures and covariates

In line with the clinical assessment framework described above, six outcome measures were selected for analyses. In the linear mixed-effects models, side, age, and sex were included as fixed effects to adjust for factors that could potentially influence finger tapping performance. Because each participant contributed repeated measurements under both eyes-open and eyes-closed conditions, the subject was included as a random intercept to account for the within-subject correlation.

### 2.7 Statistical analysis

The primary objective of this study was to reveal whether and how the effect of eye closure on finger tapping performance differs between the PD and HC groups. For each outcome metric, the logarithmic ratio between the eyes-open and eyes-closed conditions was computed as follows:

$$r=\ln(\frac{X_{\text{Closed}}}{X_{\text{Open}}})$$

This logarithmic ratio was used to quantify the eye-closure effect as a proportional change relative to the eyes-open condition, while reducing the influence of interindividual differences in absolute values. In addition, this transformation represents increases and decreases on a symmetric scale, with positive values indicating larger values under eyes-closed conditions and negative values indicating smaller values under eyes-closed conditions. The logarithmic ratio served as the response variable in the linear mixed-effects model. The model included group (PD vs. HC), side (left vs. right), age, and sex as fixed effects and subject as a random intercept:

$$r_{\mathrm{ij}}=\beta_{0}+\beta_{1}{\text{Group}}_{i}+\beta_{2}{\text{Side}}_{j}+\beta_{3}{\mathrm{Age}}_{i}+\beta_{4}{\mathrm{Sex}}_{i}+b_{i}+\varepsilon_{\mathrm{ij}}$$

Where $b_{i}∼N(0,\sigma_{b}^{2})$ and $\varepsilon_{\mathrm{ij}}∼N(0,\sigma^{2})$ . The main parameter of interest was the Group effect (β₁), representing the between-group difference in the eye-closure effect. Regression coefficients, 95% confidence intervals, and *p*-values are reported.

The de-identified dataset used for these analyses (tapping_trial_metrics.csv), containing one row per 10 s tapping trial (subject × side × eye condition) with Subject ID, group, age, sex, side, eye condition, Tapping Count, and the six outcome measures, is provided in Supplementary File S2.

To characterize the baseline differences between the groups, we compared age, sex, and handedness between patients with PD and HC. Age was compared using the Mann–Whitney U test. Sex (female/male) distributions were compared between the groups using the chi-square test. Handedness (right/left-dominant) was compared using Fisher’s exact test because of the small expected cell counts. To further characterize baseline motor performance during the task, eyes-open finger-tapping outcomes were compared between the PD and HC groups separately for the left and right hands. Group comparisons were performed using Welch’s *t*-test.

All statistical analyses were conducted using JMP® Student Edition 19.0.1 (Build 887,995; SAS Institute, Cary, NC, USA) on Microsoft Windows 11 Pro.

## 3 Results

### 3.1 Demographic and clinical characteristics

Table 1 summarizes the demographic and clinical characteristics of the participants. There were no significant differences between the PD and HC groups in terms of age, sex, or handedness (all *p* > 0.050).

**Table 1:** Demographic and clinical characteristics of the participants.

Variable	PD (*n* = 45)	HC (*n* = 20)	*p*-value
Age, years	74.0 ± 6.6	74.6 ± 5.5	0.79
Female, *n* (%)	21 (46.7)	13 (65.0)	0.17
Right-handed, *n* (%)	43 (95.6)	20 (100.0)	1.00
PD clinical characteristics			
Age at onset, years	68.5 ± 7.8	—	—
Disease duration, years	5.4 ± 3.5	—	—
Hoehn & Yahr stage, *n* (%)			
II	41 (91.1)	—	—
III	4 (8.9)	—	—
MDS-UPDRS Part III total	28.9 ± 10.6	—	—
Finger tapping (right), *n* (%)			
0	2 (4.4)	—	—
1	14 (31.1)	—	—
2	28 (62.2)	—	—
3	1 (2.2)	—	—
Finger tapping (left), *n* (%)			
0	3 (6.7)	—	—
1	9 (20.0)	—	—
2	30 (66.7)	—	—
3	3 (6.7)	—	—
LEDD, mg	741.8 ± 348.9	—	—

Data are presented as mean ± SD or n (%). The *p*-values indicate group comparisons between PD and HC: Mann–Whitney U test for age, chi-square test for sex, and Fisher’s exact test for handedness. Clinical characteristics are shown only for the PD group; therefore, no between-group statistical comparisons were performed for these variables. Finger tapping scores were assessed using Part III, Item 3.4 (Finger Tapping) of the MDS-UPDRS. LEDD, levodopa-equivalent daily dose.

### 3.2 Comparison of eye-closure effects on finger-tapping performance

Tables 2, 3 present the outcome measures in the PD and HC groups, respectively, under eyes-open and eyes-closed conditions. Under eyes-open conditions, patients with PD showed nominally lower mean amplitude and mean velocity and nominally higher interval variability than HCs in both the left and right hands (Supplementary Tables S1.1, S1.2). Velocity variability was also nominally lower in patients with PD than in HCs in both hands. In contrast, mean interval and amplitude variability did not show nominal group differences. These exploratory baseline comparisons suggest that, even under eyes-open conditions, patients with PD showed reduced movement amplitude and velocity and less stable tapping intervals compared with HCs.

**Table 2:** Outcome measures in the PD group.

Outcome measure	OPEN-R	OPEN-L	CLOSE-R	CLOSE-L
Mean amplitude (rad)	0.448 ± 0.173	0.466 ± 0.196	0.408 ± 0.193	0.407 ± 0.204
Amplitude variability (rad)	0.101 ± 0.050	0.095 ± 0.035	0.088 ± 0.033	0.091 ± 0.037
Mean velocity (rad/s)	2.629 ± 0.762	2.511 ± 0.855	2.269 ± 0.824	2.128 ± 0.784
Velocity variability (rad/s)	3.417 ± 0.939	3.238 ± 1.066	2.989 ± 1.033	2.800 ± 0.989
Mean interval (s)	0.323 ± 0.084	0.357 ± 0.110	0.337 ± 0.109	0.364 ± 0.127
Interval variability (s)	0.033 ± 0.025	0.034 ± 0.017	0.037 ± 0.030	0.041 ± 0.034

**Table 3:** Outcome measures in the HC group.

Outcome measure	OPEN-R	OPEN-L	CLOSE-R	CLOSE-L
Mean amplitude (rad)	0.610 ± 0.180	0.654 ± 0.138	0.546 ± 0.201	0.583 ± 0.137
Amplitude variability (rad)	0.102 ± 0.040	0.108 ± 0.041	0.092 ± 0.043	0.103 ± 0.034
Mean velocity (rad/s)	3.640 ± 0.954	3.852 ± 0.933	3.247 ± 1.033	3.538 ± 1.067
Velocity variability (rad/s)	4.686 ± 1.160	4.874 ± 1.112	4.195 ± 1.256	4.501 ± 1.235
Mean interval (s)	0.322 ± 0.057	0.330 ± 0.059	0.321 ± 0.059	0.327 ± 0.060
Interval variability (s)	0.019 ± 0.005	0.025 ± 0.008	0.017 ± 0.005	0.022 ± 0.008

Values are presented as the mean ± standard deviation. OPEN, eyes open; CLOSE, eyes closed; R, right; L, left.

Table 4 shows the results of the linear mixed-effects models used to compare the effects of eye closure between the two groups. For each of the six outcome measures, the effect of eye closure was quantified as the logarithmic ratio between the eyes-closed and eyes-open conditions, r=ln(Closed/Open) . A nominal group effect was observed only for interval variability (*p* = 0.0337), whereas no group differences were observed for the other outcome measures.

**Table 4:** Group effects (HC–PD) on the log eye-closure ratios for six finger-tapping outcome measures.

Outcome measures	Group effect (*β*)	SE	95% CI lower bound	95% CI upper bound	*p*-value
Mean amplitude (rad)	0.0059	0.047	−0.089	0.101	0.9016
Amplitude variability (rad)	0.0004	0.062	−0.124	0.125	0.9951
Mean velocity (rad/s)	0.0420	0.043	−0.043	0.127	0.3274
Velocity variability (rad/s)	0.0299	0.038	−0.046	0.105	0.4308
Mean interval (s)	−0.0315	0.017	−0.066	0.003	0.0739
Interval variability (s)	−0.1859	0.086	−0.357	−0.015	0.0337*

Values are regression coefficients (β) for the Group effect (HC–PD contrast) from linear mixed-effects models with r = ln(Closed/Open) as the response. Each model included group (PD vs. HC), side (left vs. right), Age, and Sex as fixed effects and the subject as a random intercept. A positive β value indicates a larger eye-closure effect in the HC group than in the PD group. SE, standard error; CI, confidence interval. *Nominal *p* < 0.05.

In additional within-group analyses using left–right averaged raw values, interval variability showed a nonsignificant numerical increase in the PD group (+15.4%; *p* = 0.077) and a nonsignificant numerical decrease in the HC group (−11.3%; *p* = 0.081) (Supplementary Table S2). Across all 260 test trials, the median Tapping Count per 10-s trial was 29 (interquartile range, 25–35; range, 11–50), corresponding to a median of 28 inter-peak intervals (interquartile range, 24–34; range, 10–49) available for estimating interval variability. Trial-level Tapping Counts are provided in Supplementary File S2 (tapping_trial_metrics.csv). The distribution of interval variability under each condition is shown in Supplementary Figure S1.

## 4 Discussion

In this study, we examined whether visual information contributes to compensatory control of finger-tapping performance in PD by comparing eyes-open and eyes-closed conditions in patients with PD and HCs. A nominal between-group difference in the eye-closure effect was observed for interval variability in this exploratory analysis. In additional within-group analyses, interval variability showed a nonsignificant numerical increase in the PD group and a nonsignificant numerical decrease in the HC group. These opposing numerical trends raise the possibility that the effect of eye closure on the stability of continuous tapping rhythms may differ between the groups and warrant further investigation.

Previous studies have shown that visual feedback and external visual cues can influence motor performance in patients with PD. For example, restricting visual feedback worsens performance during upper limb reaching and gait-related tasks, whereas visual information or external visual cues can facilitate movement execution (15–17). Adamovich et al. (16) reported that patients with PD were selectively less accurate than controls when pointing required the integration of arm proprioception with concurrent or remembered visual target information, whereas mean endpoint accuracy was preserved when visual feedback of the moving finger was available. Keijsers et al. (18) further suggested that deficits in proprioceptive information processing may occur early in PD and may be partially compensated by visual feedback in milder disease, although the benefit of visual feedback may decrease with disease progression. These findings suggest that visual input may provide an external reference for monitoring body position and guiding movement and may partly compensate for impaired proprioceptive processing in PD. However, these studies mainly focused on reaching and gait-related tasks. Our results add to the literature by suggesting that visual input may also influence temporal regularity during a simple and commonly used finger-tapping task for assessing bradykinesia, and that this influence may differ between patients with PD and HCs.

Our findings may provide mechanistic clues regarding how visual input interacts with rhythmic stability during repetitive movement. In healthy individuals, temporal regularity during continuous tapping does not necessarily depend on visual feedback (19). Moreover, eye closure enhances somatosensory perception, including proprioception (20). The numerical decrease in interval variability observed in HCs under eyes-closed conditions in this study may suggest that removing visual input reduces visual distraction and allows for more stable internal or somatosensory-guided timing. In contrast, the numerical increase in interval variability observed in patients with PD may suggest that temporal regularity becomes more vulnerable when visual input is reduced, potentially because of greater reliance on proprioceptive processing.

These opposing numerical trends may help explain why the group difference was most apparent for interval variability. Amplitude, velocity, and mean interval are closely related to the instruction to tap as widely and as quickly as possible, and both patients with PD and HCs may have used visual feedback to adjust these aspects of performance under eyes-open conditions. When visual input was removed, the effect on these measures may have been relatively similar between the groups. In contrast, interval variability reflects the stability of rhythmic timing across successive taps; therefore, the divergent response to eye closure between HCs and patients with PD may have been expressed most clearly in this measure. However, these mechanistic interpretations remain speculative because visual dependence, motor automaticity, attentional compensation, proprioception, and sensory integration were not directly measured.

Clinically, this opposing numerical pattern may warrant further investigation as a potential supportive diagnostic feature. Under eyes-open conditions, the vulnerability of internal timing in patients with PD may be partially masked by visual compensation. Under eyes-closed conditions, this compensation may be reduced and temporal instability may become more apparent. Accordingly, evaluating the tapping rhythm during eye closure may provide a simple approach for assessing impaired automatic control in PD. Because this approach can be implemented using an extremely simple task, it may be feasible for use in routine clinical settings if its diagnostic value is confirmed. However, diagnostic performance was not evaluated in the present study, and its clinical utility and sensitivity, particularly in early-stage PD, should be further assessed in future studies.

This study had several limitations. First, the relatively small sample size limited the assessment of clinical heterogeneity, cognitive function, and visuospatial characteristics. Second, only one test trial was performed per condition, with a fixed order (eyes open before eyes closed; right before left); thus, trial variability and practice, fatigue, or order effects, which may differ between groups, may have confounded the eye-closure effect. In addition, interval variability was estimated from inter-peak intervals within a single 10-s trial and may therefore have been sensitive to sampling variability, particularly in trials with fewer taps. Future studies should use repeated trials and randomized or counterbalanced task orders. Third, the wearable finger-tapping device and software pipeline were not formally validated against blinded clinical ratings or quantitative video-based analyses. Finally, because this study was exploratory, no formal correction for multiple comparisons was applied. Thus, the nominal group effect observed for interval variability should be interpreted with caution and confirmed in larger studies with interval variability prespecified as the primary outcome.

## 5 Conclusion

The present exploratory findings raise the possibility of a PD-specific effect of visual input on rhythmic stability during finger tapping. The opposite numerical effects of eye closure in the PD and HC groups are consistent with the possibility that visual information partially compensates for reduced motor automaticity in PD. These findings suggest that eyes-closed finger tapping may help reveal altered temporal motor control in PD and that this approach warrant further investigation as a potential supportive diagnostic tool. Future studies are needed to evaluate its potential diagnostic utility, confirm the robustness of this effect, and further investigate its relationship with clinical disease severity and cue-responsive motor phenomena, such as freezing of gait.

## Data Availability

The original contributions presented in the study are included in the article/Supplementary material, further inquiries can be directed to the corresponding author.

## Footnotes

1
https://www.python.org

2
https://numpy.org

3
https://scipy.org

4
https://pandas.pydata.org

## Supplementary material

SUPPLEMENTARY File S1 Python script for computing the six tapping outcome measures from raw time–angle data (tapping_analysis_core.py).

SUPPLEMENTARY File S2 Dataset of all 10-s tapping trials used for statistical analyses (tapping_trial_metrics.csv; one row per Subject × Side × Eye condition, including Subject ID, group, age, sex, side, eye, Tapping Counts, and six outcome measures).

SUPPLEMENTARY File S3 Documentation for the code and dataset (README.md).
