# Supplementary material for: Eyes-closed finger-tapping task for assessing visual dependence in temporal motor control in Parkinson’s disease
Source: Front Neurol. 2026 Sep 29;17:1887146. doi: 10.3389/fneur.2026.1887146 (PMC13658063; doi:10.3389/fneur.2026.1887146)
Supplement: Supplementary file 1 [file Supplementary_file_4.DOCX]

| **Outcome** | **PD, mean ± SD** | **HC, mean ± SD** | **p-value** |
| --- | --- | --- | --- |
| Mean amplitude (rad) | 0.448 ± 0.173 | 0.610 ± 0.180 | 0.002* |
| Amplitude variability (rad) | 0.101 ± 0.050 | 0.102 ± 0.040 | 0.987 |
| Mean velocity (rad/s) | 2.629 ± 0.762 | 3.640 ± 0.954 | <0.001* |
| Velocity variability (rad/s) | 3.417 ± 0.939 | 4.686 ± 1.160 | <0.001* |
| Mean interval (s) | 0.323 ± 0.084 | 0.322 ± 0.057 | 0.964 |
| Interval variability (s) | 0.0335 ± 0.0247 | 0.0192 ± 0.0053 | <0.001* |

**Supplementary Table S1.1. Comparison of baseline finger-tapping performance under eyes-open conditions in the right hand.**

**Supplementary Table S1.2. Comparison of baseline finger-tapping performance under eyes-open conditions in the left hand.**

| **Outcome** | **PD, mean ± SD** | **HC, mean ± SD** | **p-value** |
| --- | --- | --- | --- |
| Mean amplitude (rad) | 0.466 ± 0.196 | 0.654 ± 0.138 | <0.001* |
| Amplitude variability (rad) | 0.095 ± 0.035 | 0.108 ± 0.041 | 0.226 |
| Mean velocity (rad/s) | 2.511 ± 0.855 | 3.852 ± 0.933 | <0.001* |
| Velocity variability (rad/s) | 3.238 ± 1.066 | 4.874 ± 1.112 | <0.001* |
| Mean interval (s) | 0.357 ± 0.110 | 0.330 ± 0.059 | 0.204 |
| Interval variability (s) | 0.0337 ± 0.0168 | 0.0248 ± 0.0084 | 0.006* |

Values are presented as mean ± standard deviation. Group comparisons between patients with Parkinson’s disease (PD) and healthy controls (HCs) were performed using Welch’s t-test. P-values are nominal and were not adjusted for multiple comparisons. Asterisks indicate nominal p < 0.05. Under eyes-open conditions, patients with PD showed nominally lower mean amplitude and mean velocity and nominally higher interval variability than HCs in both the right and left hands (Supplementary Tables S1.1 and S1.2). Velocity variability was also nominally lower in patients with PD than in HCs in both hands. In contrast, mean interval and amplitude variability did not show nominal group differences. These exploratory baseline comparisons suggest that, even under eyes-open conditions, patients with PD showed reduced movement amplitude and velocity and less stable tapping intervals compared with HCs.

**Supplementary Table S2. Within-group eye-closure effects on interval variability**

| Group | Open mean ± SD | Closed mean ± SD | Closed − Open | % change | p-value |
| --- | --- | --- | --- | --- | --- |
| PD | 0.0336 ± 0.0161 | 0.0387 ± 0.0246 | +0.0052 | +15.4% | 0.077 |
| HC | 0.0220 ± 0.0050 | 0.0195 ± 0.0052 | −0.0025 | −11.3% | 0.081 |

Values are presented as mean ± standard deviation. Interval variability was averaged across the left and right hands for each participant. Differences were calculated as eyes-closed minus eyes-open values. P-values were obtained using paired t-tests. These analyses were exploratory and were performed to describe within-group patterns.

**Supplementary Figure S1. Distribution of interval variability under eyes-open and eyes-closed conditions.**

Box-and-whisker plots of interval variability (s) for each group [healthy controls (HC), Parkinson’s disease (PD)], side (left/right), and visual condition (eyes-open vs. eyes-closed). Boxes indicate the interquartile range, horizontal lines indicate the median, and whiskers indicate the range excluding outliers. Dots indicate outliers. This pattern is consistent with the opposite numerical directions of change in temporal regularity observed between the two groups.
